# Intragraft immune cells: accomplices or antagonists of recipient-derived macrophages in allograft fibrosis?

**DOI:** 10.1007/s00018-023-04846-0

**Published:** 2023-07-03

**Authors:** Xiaoping Li, Jing Wu, Shan Zhu, Qiuyu Wei, Liyan Wang, Jingtao Chen

**Affiliations:** 1 Cancer Center, First Hospital of Jilin University, Changchun, 130021 Jilin China; 2 Laboratory for Tumor Immunology, First Hospital of Jilin University, Changchun, 130061 Jilin China; 3 Department of Pediatrics, First Hospital of Jilin University, Changchun, 130021 Jilin China

**Keywords:** Monocytes, M2 macrophages, Allotransplantation, Extracellular matrix, Colony-stimulating factor 1, Interleukin-22

## Abstract

Organ fibrosis caused by chronic allograft rejection is a major concern in the field of transplantation. Macrophage-to-myofibroblast transition plays a critical role in chronic allograft fibrosis. Adaptive immune cells (such as B and CD4^+^ T cells) and innate immune cells (such as neutrophils and innate lymphoid cells) participate in the occurrence of recipient-derived macrophages transformed to myofibroblasts by secreting cytokines, which eventually leads to fibrosis of the transplanted organ. This review provides an update on the latest progress in understanding the plasticity of recipient-derived macrophages in chronic allograft rejection. We discuss here the immune mechanisms of allograft fibrosis and review the reaction of immune cells in allograft. The interactions between immune cells and the process of myofibroblast formulation are being considered for the potential therapeutic targets of chronic allograft fibrosis. Therefore, research on this topic seems to provide novel clues for developing strategies for preventing and treating allograft fibrosis.

## Introduction

Although many patients with organ failure have been successfully treated since the development of allotransplantation, the 10-year survival rate after transplantation has not significantly improved [1, 2]. At present, allograft fibrosis caused by chronic allograft rejection is a major factor affecting the long-term survival of patients after transplantation [3, 4]. Persistent allogeneic immune-mediated inflammation under chronic dysfunction of transplanted organs leads to graft fibrosis [5–7]. However, the specific immunological mechanism of allograft fibrosis remains unclear.

It is traditionally believed that chronic allograft rejection is primarily mediated by indirect CD4^+^ T cell reaction [8]. Moreover, immunosuppressants currently used in clinical practice, which mainly act on the immune response of CD4^+^ T cells, have achieved some success; however, the long-term survival rate of grafts after organ transplantation must be improved. It has also been reported that innate immune cells can initiate the rejection of allogeneic nonself independent of lymphocytes [9–12], and an increasing number of recent and ongoing studies have focused on innate immune cells [13, 14]. Further, neutrophils, monocyte-derived dendritic cells (mo-DCs), plasmacytoid dendritic cells (pDCs), and innate lymphoid cells (ILCs) reportedly participate in chronic allograft rejection during mouse heart transplantation [15–17], mouse bone marrow plug transplantation [12], graft-versus-host disease (GVHD) [18], and mouse lung, skin, and human intestinal transplantation [19–21], respectively. It is reported that knocking out or inhibiting the function of recipient-derived macrophages after mouse lung, heart, and kidney allotransplantation reduce chronic allograft rejection [9, 22, 23].

Allograft immune rejection results from the coordinated response of several immune cell types as well as a series of complex processes in which the innate immune systems of the donor and recipient interact with the adaptive immune system. In this review, the latest progress of research on the interaction between recipient-derived macrophages and other immune cells, as well as their role in allograft fibrosis following chronic allograft rejection was reviewed. Furthermore, the molecular biological mechanism of recipient-derived macrophage plasticity in chronic allograft rejection was examined. This work provides a theoretical basis for developing targeted drugs to prevent and treat allograft fibrosis.

## Plasticity of recipient-derived macrophages in allograft fibrosis

Peripheral blood monocytes can undergo epigenetic changes according to local tissue microenvironment changes [24]. When entering tissues, monocytes can differentiate into either monocyte-derived macrophages (mo-Macs) or mo-DCs [25]. The transcription factors involved in CD14^+^ monocyte differentiation are categorized as either mo-Mac-expressed (MafB, PU.1) or mo-DC-expressed (IRF4, AHR) [26]. mo-Macs express MafB and moderate levels of PU.1, which have been found to inhibit mo-DC differentiation [27]. In the presence of colony-stimulating factor 1 (CSF-1), CD14^+^ monocytes differentiate into mo-Macs unless they are exposed to certain cytokines (i.e., IL-4 and TNFα) in conjunction with AHR ligands, which promote mo-DC differentiation [26]. However, allograft immunity research has not yet elucidated the mechanism by which the recipient monocytes transform into macrophages and their various subsets in the allograft.

Allogeneic nonself-recognition by monocytes is necessary to initiate graft rejection. Dai et al. found for the first time that the paired immunoglobulin-like receptor-A (PIR-A) expressed on recipient Ly6C^high^ monocytes and macrophages promote allograft rejection via direct binding to donor MHC I molecules, and inhibiting PIR-A recognition of MHC I using a specific antibody attenuates kidney and heart allograft rejection in mice [9]. Monocyte activation after allotransplantation can also occur independently of MHC mismatch between donors and recipients [10, 28]. Donor polymorphism in the gene encoding signal regulatory protein α (SIRPα) can regulate the affinity with CD47 on recipient Ly6C^high^ monocytes to accumulate mature mo-DCs that produce interleukin-12 and present antigens to T cells [12, 29], which appears to aggravate organ rejection after allotransplantation. However, blocking the binding of the recipient monocyte SIRPα to CD47 from donor cells impairs transplant tolerance [30], which results in the differentiation of the donor myeloid-derived suppressor cells into M1 macrophages [31]. Therefore, it is possible that the strength of the interaction between SIRPα expressed by recipient or donor monocytes and CD47 expressed by donor or recipient cells determines whether monocyte differentiation favors mo-Macs or mo-DCs following allotransplantation.

The accumulation of macrophages in grafts has long been identified as a characteristic of organ transplantation rejection [32–34]. Immunological examination of grafts with chronic rejection showed that macrophages accounted for 74.2–86.6% of the total immune cells [6, 35]. Approximately 90% of macrophages in grafts were derived from monocytes obtained from recipient bone marrow [22, 23]. Recipient-derived macrophages have different characteristics from those of resident donor tissue macrophages [36]. These resident macrophages are sensitive to the stimulation of the transplantation environment and are prone to necrosis and apoptosis, while recipient-derived macrophages continuously replenish the lost resident macrophages in the graft [37–39]. Notably, this process persists after the acute surgical inflammation subsides [40]. Moreover, recipient-derived macrophages have considerable plasticity [41], and may thus play an important role in long-term immune rejection after allotransplantation.

Myofibroblasts [α smooth muscle actin^+^ (α-SMA^+^) fibroblasts] can produce extracellular matrix (ECM) components, which are the primary effectors of the organ fibrotic process in chronic allograft rejection [22, 23, 42, 43]. Myofibroblasts may arise from resident fibroblasts, pericytes, endothelial and epithelial cells, and circulating precursors [44–47]. Recent studies have primarily focused on myofibroblasts derived from recipient bone marrow progenitor cells [22, 23]. Macrophages derived from bone marrow cells can differentiate into α-SMA^+^ myofibroblasts within a fibrotic tissue in a process termed macrophage-to-myofibroblast transition (MMT) [22, 23, 41, 48–51]. To determine the origin of myofibroblasts in models of chronic allograft rejection, lineage tracing using LysM^Cre^Rosa26-tdTomato and CX3CR1^creER^Rosa26-tdTomato mice has been employed, and to track myeloid cells and confirm the potential sources of α-SMA^+^ myofibroblasts, allografts have been analyzed by confocal microscopy, flow cytometry, and single-cell transcriptomic analysis to identify co-expression of macrophage makers (CX3CR1, CD68, or F4/80) and the myofibroblast marker (α-SMA) as well as collagen production [22, 23, 43]. MMT may serve as a key checkpoint for the progression of chronic allograft fibrosis.

Recipient monocytes infiltrate into the allograft and are mainly polarized into M1 macrophages and M2 macrophages [52, 53]. In general, IFNγ/LPS/TNFα promote M1 macrophage activation (phenotypic markers: MHC II, CD86, Ly6c^high^, and iNOS), while IL-4/IL-10/IL-13/TGFβ/CSF-1 stimulate M2 macrophage activation (phenotypic markers: CD206, Ly6c^low^, CX3CR1^high^, and Arg1) [17, 23, 54, 55]. M1 macrophages tend to produce IL-1, IL-6, IL-12, IL-23, CCL2, CXCL9, CXCL10, and CXCL11, which are involved in acute rejection [54, 56]. M2 macrophages produce fewer cytokines, such as IL-10 and TGFβ1, to regulate tissue inflammation resolution and produces pro-fibrotic mediators, such as CCL18 [57] and ECM, which promotes the progression of allograft fibrosis [3, 23, 43]. The infiltration of M2 macrophages is especially related to tissue fibrosis [3, 58]. Therefore, M2 macrophages likely play a key role in allograft fibrosis.

In vitro and in vivo experiments have demonstrated that the Notch2 signaling pathway controls the transformation of Ly6C^high^ monocytes into Ly6C^low^ CX3CR1^high^ monocytes under homeostasis [59]. The induction of the colony-stimulating factor receptor-1(CSF-1R) signaling axis is necessary for the differentiation of Ly6C^high^ monocytes into Ly6C^low^ CX3CR1^high^ monocytes under pathological conditions [60, 61], and this is the main mechanism by which M2 macrophages proliferate and accumulate following allotransplantation [22, 62]. Smooth muscle cell-related gene expression in CD16^high^ CX3CR1^high^ monocytes is increased during chronic rejection of transplanted human kidney [63]. It has been reported that increased levels of Ly6C^low^ CX3CR1^high^ macrophages are consistent with fibrosis or gene-related M2 macrophage function [64]. The recipient-derived CX3CR1^high^ population, which is derived from infiltrating Ly6C^high^(mouse) /CD14^high^(human) monocytes, has a profibrotic function and is composed of different continuously differentiated subsets of cells [22, 41]. Recipient-derived CX3CR1^high^ macrophages may be mainly CD206^+^ M2 macrophages in chronic allograft fibrosis, and it has been reported that CD206^+^ M2 macrophages are the precursors of MMT [23, 41, 43, 50, 65, 66]. However, whether early M1 macrophages switch phenotype to become M2 macrophages remains unclear.

**Fig. 1:**
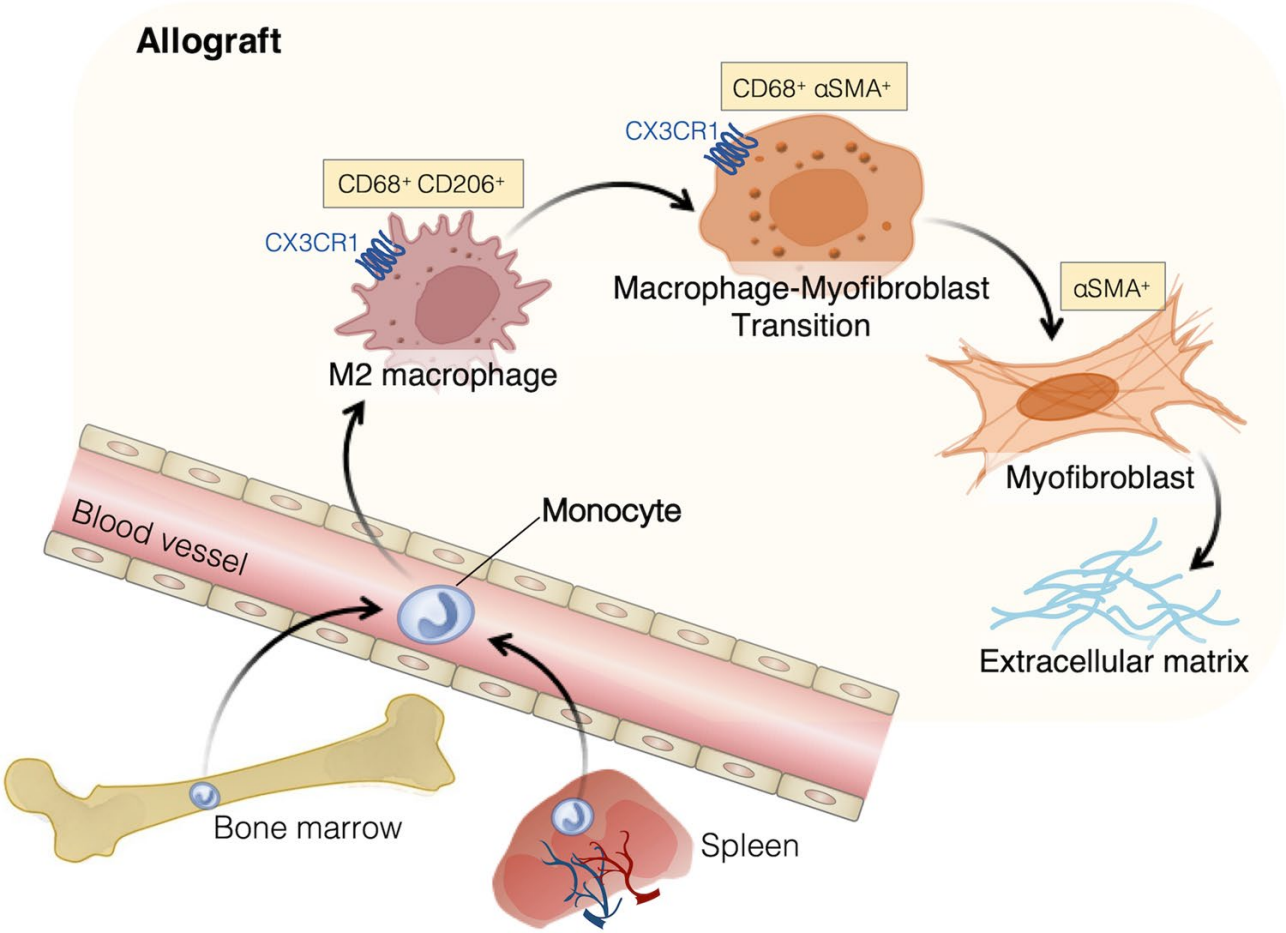
The plasticity of recipient-derived macrophages in chronic allograft rejection. Peripheral blood monocytes can undergo epigenetic changes according to the allograft microenvironment. In the allograft with chronic rejection, recipient monocytes can differentiate into CX3CR1^+^ CD68^+^ macrophages. MMT (recipient-derived CX3CR1^+^ CD68^+^ CD206^+^ M2 macrophages to α-SMA^+^ myofibroblasts) can result in the production of ECM components, which promote chronic allograft fibrosis.

The findings outlined above indicate that recipient-derived CX3CR1^high^ macrophages transformed to myofibroblasts via MMT play a critical role in chronic allograft fibrosis (Fig. 1). Understanding the dynamic reprogramming landscapes of recipient-derived macrophages is important to exploit the mechanisms of chronic allograft fibrosis. However, chronic allograft rejection does not arise from recipient-derived macrophage activity alone but from a coordinated immune cell response, and the role of recipient-derived macrophages and other immune cells in allograft fibrosis has not yet been systematically reviewed; relevant studies are discussed in the next section.

## Interaction of recipient-derived macrophages with adaptive immune cells in allograft fibrosis

### Interaction with B cells

The population of B cells has been found to increase gradually during chronic transplant rejection [2, 67, 68]. Antibody-mediated rejection (AMR) is a major determinant of chronic allograft failure [69–72]. HLA-donor-specific antibody (DSA) binding to vascular endothelial cells of the allograft triggers inflammation, vessel injury, and AMR. The histological characteristic of AMR is the accumulation of recipient-derived macrophages [73, 74]. Therefore, the interaction between macrophages and B cells likely plays an important role in chronic graft rejection.

During human chronic lung allograft dysfunction, macrophages and B cells coexist in the luminal tissue of the bronchial tub [75, 76], and the existence of DSA may promote the development of bronchiolitis obliterans syndrome after human lung allotransplantation [77]. The accumulation of CD68^+^ macrophages in endomyocardial tissue following human cardiac transplantation correlates well with the increased numbers of alloantibodies [74]. The mechanism underlying the interaction between B cells and macrophages in chronic transplant rejection requires further investigation.

In recipient mice with B cell loss, macrophage infiltration and ECM accumulation were significantly reduced and graft fibrosis was alleviated [67, 78, 79]. Thus, B cells have been demonstrated to promote allograft fibrosis. In a mouse skin graft model, activated MHC II^high^ macrophages produce B cell activating factor (BAFF), which activates B cells to secrete DSA, leading to graft damage in AMR [80]. Moreover, cardiac allografts from murine recipients treated with MHC I DSA promoted monocyte differentiation into CD68^+^ CD206^+^ M2 macrophages in the endothelium of grafts [81]. Therefore, macrophages recruit B cells in the early stage of graft rejection, while B cells secrete DSA to promote the accumulation of M2 macrophages, promoting allograft fibrosis.

**Fig. 2:**
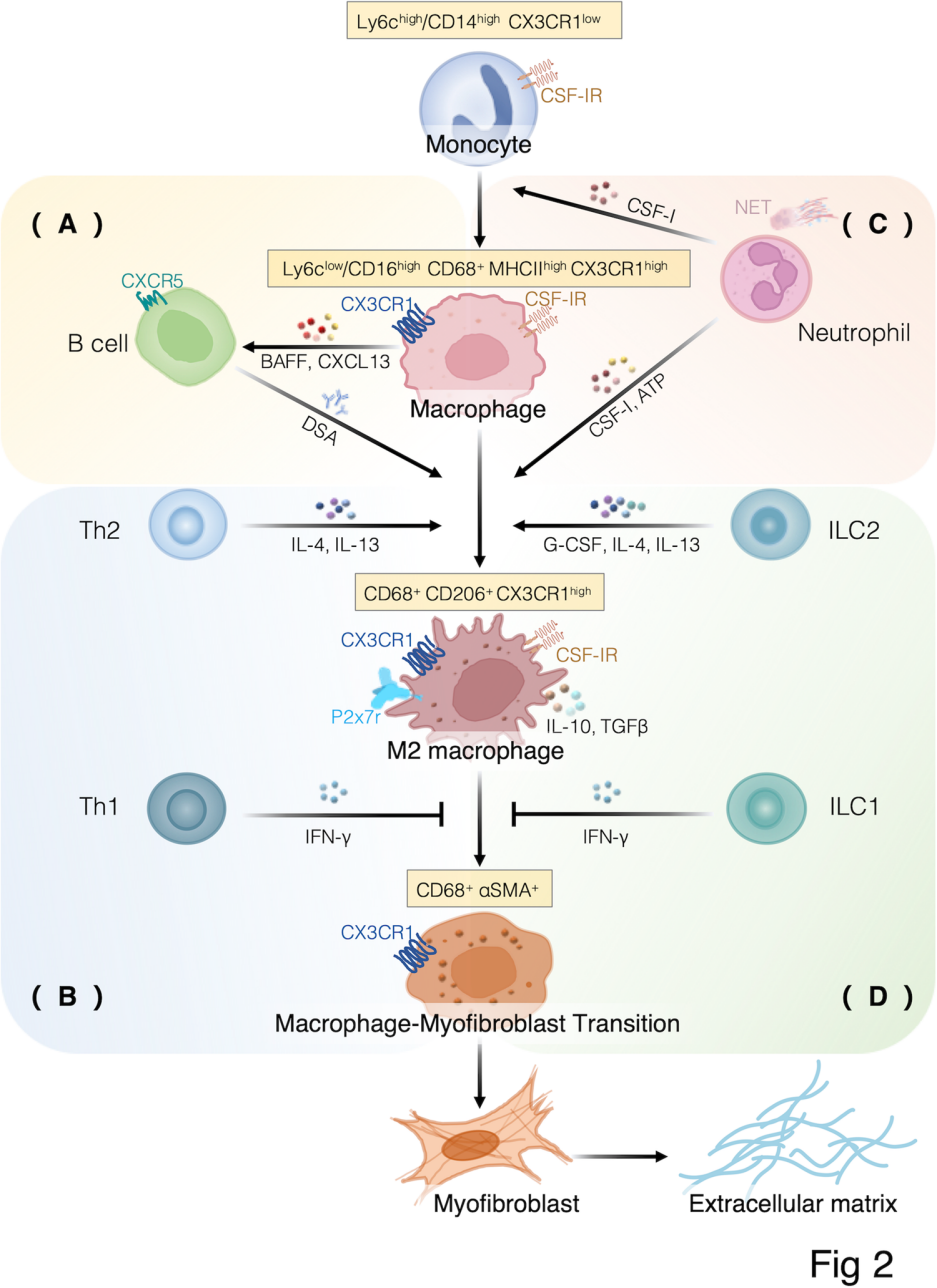
The diverse immune reactions of recipient-derived macrophages in chronic allograft rejection. Recipient-derived macrophages transformed to myofibroblasts via MMT play a critical role in chronic allograft fibrosis. During the occurrence of MMT, **A** CXCL13 and BAFF secreted by macrophages recruit and activate CXCR5^+^ B cells, which secrete DSA, which promotes the accumulation of CD68^+^ CD206^+^ M2 macrophages in grafts, while **B** IFN-γ secreted by Th1 cells inhibits the occurrence of MMT, and Th2 cells can induce the accumulation of M2 macrophages in grafts by secreting IL-4 and IL-13. **C** Neutrophils secrete CSF-1 or extracellular ATP, resulting in high M2 macrophage accumulation in the allograft. **D** IFN-γ secreted by ILC1s inhibits the occurrence of MMT, and ILC2s can induce the accumulation of M2 macrophages in grafts by secreting IL-4, IL-13, and G-CSF.

CSF-1R^+^ macrophages secrete CXCL13 to recruit CXCR5^+^ B cells to promote implant fibrosis, but the reduction in fibrosis after CSF-1R inhibitor administration was more significant than that after B cell knockout [67]. In a mouse lung transplant experiment, CXCL13 neutralization concurrent with Foxp3^+^ T cell depletion prevented AMR and preserved the airway epithelium [69]. Therefore, during chronic transplantation rejection, MHC II^high^ macrophages are responsible for CXCR5^+^ B cell recruitment and activation by secreting CXCL13 and BAFF, and activated CXCR5^+^ B cells secrete DSA, which promotes the emergence of a large number of CD68^+^ CD206^+^ M2 macrophages in the grafts (Fig. 2A). Macrophages may play a leading role in this process.

### Interaction with CD4^+^ T cells

Recipient-derived macrophages are the dominant cell population after allotransplantation [9, 10, 73]. However, human chronic lung allograft dysfunction is associated with an increase in allo-reactive CD4^+^ T cells and mononuclear phagocytic cells [82]. In fact, the fibrosis degree and M2 macrophage count could be regulated by the T cell response [83]. The ability of primed macrophages to reject allografts requires the help of CD4^+^ T cells but not of CD8^+^ T cells [84]. Therefore, the interaction between recipient-derived macrophages and CD4^+^ T cells is considered crucial in chronic allograft rejection.

The Th1 cell surface protein Tim-3 regulates macrophage activation and the severity of autoimmune diseases [85]. However, direct interaction of recipient-derived macrophages with Th1 cells has not been detected in chronic allograft rejection. Donor-derived VIP-expressing pDCs limit IFN-γ^+^CD4^+^ T cell proliferation and polarization in vitro, thereby preventing GVHD [18]. It is suggested that IFN-γ^+^CD4^+^ T cells promote chronic allograft rejection. However, Th1 cells directly promote collagen degradation by releasing IFN-γ, which is an antifibrotic cytokine [86]. Moreover, IFN-γ secreted by Th1 cells disrupts fibrosis mediated by the TGFβ/Smad3 signaling pathway [87, 88]. The transformation of recipient-derived M2 macrophages into myofibroblasts is regulated by the TGFβ/Smad3 signaling pathway [23, 51, 89]. Therefore, Th1 cells may inhibit the development of allograft fibrosis by secreting IFN-γ to inhibit MMT (Fig. 2B). This may be one of the reasons why immunosuppressants targeting CD4^+^ T cells have not been effective in preventing and treating allograft fibrosis.

Activated Th2 cells secrete IL-4 and IL-13, which can induce the accumulation of M2 macrophages and promote graft fibrosis [7, 19, 90] (Fig. 2B), and promote MMT through activation of the JAK/STAT pathway [41]. However, immunosuppressants targeting CD4^+^ T cells fail to prevent and treat allograft fibrosis. Th2 cells may not play a major role in the transformation of recipient monocytes into M2 macrophages in the allograft microenvironment. Moreover, the number and function of innate lymphoid cells (ILCs) in the recipient circulation are reportedly unaffected by immunosuppressive drugs [91]. Type 2 ILCs (ILC2s) also secrete IL-4 and IL-13 [19], which may not be affected by immunosuppressants targeting CD4^+^ T cells.

It has been reported that inhibiting proliferation and accumulation of macrophages in the graft can reduce the risk of rejection without changing the number of peripheral leukocytes or altering T cell function [62, 92]. T cell knockout did not improve the degree of fibrosis on the implant surface, while macrophage knockout almost completely improved fibrosis on the surface [67]. It has been reported that CD4^+^ T cells also promote macrophages to acquire long-term specific memory of mouse skin grafts [93]. In the process of chronic rejection, the progressive accumulation of recipient-derived macrophages may be the main mechanism leading to allograft fibrosis, and CD4^+^ T cells play an auxiliary role in this process.

## Interaction of recipient-derived macrophages with innate immune cells in allograft fibrosis

### Interaction with neutrophils

Neutrophils can promote immune rejection of allogeneic transplants. In the early stages of rejection after transplantation, activated neutrophils cause tissue damage by releasing neutrophil extracellular traps (NETs) and proteases [94, 95]. Extensive NETs were formed in the grafts of acute renal tubular necrosis after human kidney transplantation and primary graft dysfunction (PGD) after human lung transplantation [95, 96]. In addition, neutrophils can initiate early rejection by interacting with monocytes and macrophages. In transplant-mediated ischemia reperfusion injury, intravital 2P microscopy revealed that neutrophils migrate across the endothelium immediately following inflammatory Ly6C^high^ monocyte migration [97]. Neutrophils can damage cells or the ECM and release danger-associated molecular patterns (DAMPs) [98]. DAMPs then stimulate tissue-resident macrophages to secrete inflammatory mediators (such as ELR^+^-CXC and IL-1β) that activate the vascular endothelium and recruit circulating neutrophils, which adhere to the vascular endothelium of the graft [99].

Neutrophils have an important role in chronic allograft rejection [99]. A report showed that the accumulation of neutrophil-derived defensin peptides influenced the induction of airway inflammation and fibrosis in human lung allografts [100]. Christoffersson et al. found a unique vascular endothelial growth factor-A-induced subpopulation of CXCR4^high^ MMP-9^high^ neutrophils with vascular remodeling ability in mouse islet graft [101]. Macrophages, lymphocytes, and neutrophils highly infiltrated chronic rejection grafts [6, 102]. In a study of mouse heart transplantation, it was found that neutrophils were the main source of CSF-1 after co-stimulation block, which promoted the transformation of Ly6c^high^ monocytes into M2 macrophages[16] (Fig. 2C). Therefore, neutrophils may indirectly play a role in promoting fibrosis by promoting the transformation of recipient-derived macrophages.

Neutrophils in inflammatory tissues can produce elevated levels of extracellular adenosine triphosphate (ATP) [103]. The purinergic receptor P2X7 (P2x7r), an ATP-gated ion channel protein, and its main ligand, extracellular ATP, have recently been found to play an important role in promoting CD11b^+^ CD206^+^ P2x7r^+^ macrophage-mediated myocardial fibrosis during mouse chronic cardiac transplantation rejection [17] (Fig. 2C). In inflammatory tissues at the chronic graft rejection site, it remains unclear whether neutrophils produce a large number of extracellular ATP and promote the accumulation of M2 macrophages in allografts through the ATP/P2x7r axis.

Neutrophil knockout alone does not significantly improve fibrosis caused by chronic rejection [67]. In a mouse acellular nerve allograft (ANA) model, systemic depletion of recipient-derived macrophages (but not neutrophils) severely hindered angiogenesis and subsequent ANA nerve regeneration, suggesting that blood-derived macrophages were the main contributors to angiogenesis in ANAs [104]. Further investigation is required to determine whether neutrophils secrete a large amount of CSF-1 or extracellular ATP for long periods, resulting in high M2 macrophage accumulation in the allograft in chronic organ transplant rejection, thus aggravating organ fibrosis.

### Interaction with ILCs

In recent years, ILCs have been newly identified with innate cell-like characteristics [105–107] and found to play several important roles in the innate immune system [108]. ILCs are classified into three main groups: group 1 (ILC1s and NK cells), group 2 (ILC2s), and group 3 (ILC3s), which correspond to Th1 (NK cells correspond to CD8^+^ cytotoxic T cells), Th2, and Th17 cells, respectively [106]. They lack conventional antigen receptors, instead identifying non-specific danger signals and cytokines [105].

ILCs reside in a variety of tissues and can proliferate and enrich regionally[109, 110], which helps the innate immune system to quickly initiate defensive responses and participate in tissue repair and homeostasis [111]. In pathological settings, ILCs can be supplemented from bone marrow or lymphoid organ precursors, in addition to local proliferation [109, 112].

ILCs are implicated in chronic inflammation, autoimmunity, and cancer [113–116]. In the field of organ transplantation, ILCs are categorized as donor-derived or recipient-derived ILCs. In human bone marrow transplantation, a high number of donor-derived ILCs tend to proliferate and are related to GVHD development [117]. In mouse liver and heart allograft, the infiltration of recipient cells increased, and the depletion of donor lymphocytes occurred on the 28th day after transplantation. Moreover, conventional NK cells and ILC1s in grafts showed an increasing trend and were mainly derived from the recipient; however, changes in ILC2s and ILC3s have not been verified further at the late transplantation period [2]. The number and the function of ILCs in the recipient’s circulation are reportedly unaffected by immunosuppressive drugs [91]. Although ILCs only account for a small proportion of graft immune cells, they likely play an important role in chronic allograft rejection.

#### Interaction with NK cells and ILC1s

Together with ILC1s, NK cells constitute group 1 ILCs, which are characterized by their capacity to produce IFN-γ and their functional dependence on the transcription factor T-bet. Human donor lung tissue with a high level of ILC1s before transplantation showed no occurrence of PGD after transplantation [118], suggesting that ILC1s may promote early transplantation tolerance. The number of ILC1s and NK cells was reportedly decreased in the early stages of mouse heart and liver allotransplantation, but that from D28 recipients was increased [2], suggesting that recipient-derived group 1 ILCs may be involved in the chronic rejection process. The role of ILC1s and NK cells in chronic allograft fibrosis, particularly with respect to macrophages, is still not well understood.

In a human bronchial asthma model experiment, ILC1s co-cultured with alveolar macrophages (AMs) induced M1 macrophage-related gene expression [119], which may be related to the secretion of IFN-γ by ILC1s. The mechanism underlying the interaction between macrophages and ILC1s during allograft fibrosis has not been reported. ILC1s may inhibit allograft fibrosis by secreting IFN-γ to inhibit MMT (Fig. 2D). NK cells have dual immunomodulatory effects after lung, kidney, skin, and heart transplantation [120–124]. After 3 to 4 days of mouse allogeneic skin grafts, the mRNA expression of monocyte chemotactic cytokines increased, and after treatment with anti-NK1.1 antibody, the mRNA levels of these cytokines in the grafts were downregulated by nearly 70% [123]. Hence, the presence of NK cells in the graft is considered to contribute to recipient-derived macrophage accumulation.

#### Interaction with ILC2s

ILC2s express the transcription factor GATA3, mainly secrete cytokines, such as IL-4, IL-5, and IL-13, and play an important role in tissue repair [124]. ILC2s were found to decrease in the early post-transplant period after lung, heart, and liver transplantation [2, 118], which may impair the development of immune tolerance. In human islet cell transplantation, IL-10 secreted by ILC2s promoted graft tolerance [125]. At present, the mechanism underlying the role of ILC2s in chronic graft rejection is not clear.

In a human bronchial asthma model experiment, ILC2s co-cultured with AMs induced M2 macrophage-related gene expression [119], which may be related to the secretion of IL-4 and IL-13 by ILC2s. It has been reported that ILC2s secrete G-CSF in addition to IL-5, IL-13, and Areg [126]. Further, G-CSF can reportedly increase the M2 macrophage ratio during donor bone marrow transplantation and prevent the occurrence of GVHD [127]. Mast cells interact with ILC2s via the IL-33/IL-13 axis after lung transplantation to promote M2 macrophage accumulation during allograft fibrosis [19]. Hence, the secretion of IL-4, IL-13, and G-CSF by ILC2s in the allograft is considered to contribute to the massive accumulation of recipient M2 macrophages and MMT (Fig. 2D).

#### Interaction with ILC3s

ILC3s express the transcription factor RORγt; secrete IL-17, IL-22, and GM-CSF under the induction of IL-2, IL-7, IL-1β, and IL-23; induce the formation of tertiary lymphoid tissue; and influence tissue defense and inflammation responses [128–130]. ILC3s consist of two distinct subsets based on cell surface expression of natural cytotoxicity receptors (NCRs), such as NKp44 in humans and NKp46 in mice [131]. NCR^+^ ILC3s produce mainly IL-22, whereas NCR^−^ ILC3s produce IL-17 and limited amounts of IL-22 [132]. In recent years, ILC3s have been reported to promote chronic inflammation and fibrosis [105, 110, 133, 134]. Nevertheless, the role of ILC3s in chronic graft rejection remains unclear.

IL-22 is a key molecule involved in tissue repair and mucosal defense. Because IL-22R is expressed in fibroblasts and myofibroblasts, IL-22 signal transduction can promote the process of fibrosis [134, 135]. IL-22 overexpression promotes the fibroblast response to TNF and the activation of pro-inflammatory fibroblasts [136]. IL-22 promotes fibrosis by enhancing the role of TGFβ [134, 137]. In a study of mouse lung transplantation model, ILC3s were the main cell group secreting IL-22, which leads to the formation of tertiary lymphoid tissue in the allograft [138]. Increased ILC3s after human skin grafting can induce psoriasis, with 78% of ILC3s expressing IL-22 and 7–13% ILC3s expressing both IL-17 and IL-22 [20]. After human intestinal transplantation, ILC3 decreased in the early stage, and the secretion of IL-22 by NKp44^+^ ILC3s in the graft showed an increasing trend one month later [21, 139]. In particular, the proportion of ILC3s in grafts with chronic rejection was relatively high over a long period after intestinal transplantation [140]. Therefore, IL22^+ ^ILC3s may promote graft fibrosis during chronic allograft rejection.

The main effector cells of chronic allograft rejection are recipient-derived macrophages. It has been reported that CX3CR1^+^ mononuclear phagocytes maintain the proliferation and survival of ILC3s by secreting IL-23 and IL-1β and promote the secretion of IL-22 by ILC3s, thus accelerating fibroblast proliferation and fibrosis [134, 141–143]. M1 macrophages have been reported to secrete IL-23 and IL-1β to promote CXCR5 expression by ILC3s, which migrate to the lungs along the CXCR5/CXCL13 axis and promote the formation of pulmonary granuloma by secreting IL-22 and IL-17 [112]. However, the mechanism underlying the interaction between macrophages and ILC3s in the allograft microenvironment has not been reported. Although the proportion of ILC3s in immune cells is low, its function cannot be underestimated. ILC3s induce the formation of a so-called battlefield for graft rejection by promoting the formation of tertiary lymphoid tissue, and their survival is supported by macrophages.

## Conclusion and perspectives

The human immune system has not evolved to account for organ transplantation. As organ transplants constitute an artificial intervention for treating patients with end-stage diseases, immune networks do not follow a natural disease response course and instead exhibit highly specific response mechanisms. In this unique pathological microenvironment, source immune cells respond to the invasion of external cells, while recipient-derived immune cells are reprogrammed [2, 144–146]. For example, MMT occurs in the allograft, and cytokines that should not be secreted by cells are secreted in the transplant microenvironment, for example, the secretion of large amounts of CSF-1 by neutrophils after allotransplantation [16]. ILC3s secrete high levels of IL-22 after human intestinal and mouse lung allotransplantation [21, 138, 139]. Such distinct and critical immune responses constitute an interesting obstacle in organ transplant immunology.

Immunosuppressants currently used in clinical practice mainly act on the molecular before and after the immune response of CD4^+^ T cells, but the long-term survival rate of grafts after organ transplantation is still disappointing. There is currently a lack of clinical inhibitors against innate immune rejection. This review focused on chronic rejection after organ transplantation. The findings outlined above indicate that recipient-derived CX3CR1^high^ macrophages transformed to myofibroblasts via MMT play a critical role in chronic allograft fibrosis. B cells, CD4^+^ T cells, neutrophils, and ILC2s participate in the occurrence of MMT by secreting cytokines (Fig. 2). Potential strategies for inhibiting the accumulation of CX3CR1^high^ macrophages in allografts should be developed to prevent and treat allograft fibrosis. According to this review, CSF-1 is the key cytokine promoting the transformation of monocytes into recipient-derived CX3CR1^high^ macrophages in grafts. Moreover, small-molecule kinase inhibitors and neutralizing antibodies against CSF-1R suppressed the accumulation of M2 macrophages and inhibited organ fibrosis, without affecting the function of other immune cells [62, 67, 147–149]. Targeting CSF-1/CSF-1R axis may allow for a more selective method for inhibiting allograft fibrosis.

However, the phenomenon of the secretion of a large amount of IL-22 by ILC3s during chronic transplant rejection cannot be ignored. IL-22 promotes fibrosis by enhancing the role of TGFβ [134] and may be involved in the proliferation of myofibroblasts and the occurrence of MMT. It has been reported that IL-22 can inhibit rejection in the early stage after transplantation [138]. However, in the late stage of chronic transplantation rejection, IL-22^+ ^ILC3 may have a fibrotic effect on transplanted organs, which needs to be proved through a large number of studies. In the future, the proportion of ILC3s or levels of IL-22 in the peripheral blood or near allografts can be measured to determine the time of initiation of organ fibrosis and the time to administer anti-IL-22 drugs. This holds promise for the prevention and treatment of fibrosis after organ transplantation.

## Data Availability

Not applicable.
